# A coding polymorphism in matrix metalloproteinase 9 reduces risk of scarring sequelae of ocular *Chlamydia trachomatis *infection

**DOI:** 10.1186/1471-2350-7-40

**Published:** 2006-04-27

**Authors:** Angels Natividad, Graham Cooke, Martin J Holland, Matthew J Burton, Hassan M Joof, Kirk Rockett, Dominic P Kwiatkowski, David CW Mabey, Robin L Bailey

**Affiliations:** 1 London School of Hygiene & Tropical Medicine, London University, London, UK; 2 Wellcome Trust Centre for Human Genetics, University of Oxford, Oxford, UK; 3 Medical Research Council Laboratories, Fajara, The Gambia

## Abstract

**Background:**

Trachoma, an infectious disease of the conjunctiva caused by *Chlamydia trachomatis*, is an important global cause of blindness. A dysregulated extracellular matrix (ECM) proteolysis during the processes of tissue repair following infection and inflammation are thought to play a key role in the development of fibrotic sequelae of infection, which ultimately leads to blindness. Expression and activity of matrix metalloproteinase 9 (MMP-9), a major effector of ECM turnover, is up-regulated in the inflamed conjunctiva of trachoma subjects. Genetic variation within the *MMP9 *gene affects *in vitro MMP9 *expression levels, enzymatic activity and susceptibility to various inflammatory and fibrotic conditions.

**Methods:**

We genotyped 651 case-control pairs from trachoma endemic villages in The Gambia for coding single nucleotide polymorphisms (SNPs) in the *MMP9 *gene using the high-throughput Sequenom^® ^system. Single marker and haplotype conditional logistic regression (CLR) analysis for disease association was performed.

**Results:**

The Q279R mutation located in exon 6 of *MMP9 *was found to be associated with lower risk for severe disease sequelae of ocular *Chlamydia trachomatis *infection. This mutation, which leads to a nonsynonymous amino-acid change within the active site of the enzyme may reduce MMP-9-induced degradation of the structural components of the ECM during inflammatory episodes in trachoma and its associated fibrosis.

**Conclusion:**

This work supports the hypothesis that MMP-9 has a role in the pathogenesis of blinding trachoma.

## Background

Trachoma, a chronic keratoconjunctivitis caused by *Chlamydia trachomatis*, is the commonest infectious cause of blindness. The blinding complications of trachoma are due to progressive scarring of the conjunctiva (trachomatous scarring) eventually leading to in-turning of eyelashes (trichiasis) and corneal opacification. Genital *C. trachomatis *infection causes similar lesions in the female genital tract contributing to ectopic pregnancy and infertility. Severe and persistent inflammation triggered by repeated conjunctival infections is believed to increase the risk of pathological scarring later in life[1].

The mechanisms of disease pathology are not completely understood. Some evidence suggests that the dysregulated ECM proteolysis seen during the processes of tissue repair following infection and inflammation [2] may play a key role in the development of fibrotic sequelae of chlamydial infection in humans. In support of this hypothesis, we have recently shown that ocular *C. trachomatis *infection upregulates the expression of MMP-9 in the human conjunctival epithelium [3]. MMP-9 activity has been detected in immune cells present in the inflammatory infiltrate in conjunctival biopsy specimens from individuals with active trachoma [4]. In addition recent comparative studies of the role of MMP-9 in genital *Chlamydia muridarum *(MoPn) infection found greater MMP-9 transcription and activity during infection in those mouse strains exhibiting increased susceptibility to fibrotic sequelae following infection [5,6].

Matrix metalloproteinases (MMPs) are a tightly regulated family of zinc-dependent enzymes that degrade structural proteins of the ECM and basement membranes. Among them, MMP-9 is a major effector of ECM turnover during homeostasis and pathology [7]. *MMP9 *expression is regulated at the transcriptional level in response to pro-inflammatory cytokines such as tumor necrosis factor (TNF) and interleukin 1 beta (IL-1β) [8]. Post-transcriptional regulation also occurs by control of activation of the secreted pro-enzyme (proMMP-9), and inhibition of proMMP-9 and MMP-9 by tissue inhibitors (TIMPs) [7].

A number of SNPs have been identified in regulatory and coding regions of the *MMP9 *gene. Some of them have been reported to affect *in vitro MMP9 *expression levels, enzymatic activity and susceptibility to various inflammatory and fibrotic conditions [9]. We tested the hypothesis that genetic variation in coding regions of *MMP9 *affects the risk of scarring sequelae of trachoma.

## Methods

### Patients

One thousand three hundred and fifteen subjects identified by clinical examination using World Health Organization (WHO) criteria were recruited from trachoma endemic villages in The Gambia. They included 651 subjects with scarring trachoma (TS), of whom 307 additionally had trichiasis (TT), and pair-matched by sex, age, ethnic group and village of residence individuals with normal eyelids. The subjects were otherwise healthy. We have previously studied and reported polymorphism at the *IFNγ *and *IL10 *loci in these subjects [10]. The study and its procedures were approved by the Gambia Government/MRC Ethics Committee (SCC 729/857), the Ethics committees of the London School of Hygiene and Tropical Medicine and of Oxford University, and are in accordance with the Declaration of Helsinki. Subjects diagnosed with trichiasis were offered free corrective surgery.

### DNA extraction and SNP genotyping

Genomic DNA was isolated from either venous blood in EDTA or buccal brush samples and genotypes were determined by the Sequenom^® ^system as previously described [10]. Primer sequences are available on request.

### Analytical methods

#### Haplotype reconstruction and frequencies

Haplotypes were inferred from population genotype data and their frequencies were estimated as previously described [10].

#### Association analysis

A crude analysis (crude Mantel-Haenzsel) was first carried out using chi-square testing to test for differences in allele and haplotype frequencies between cases of TS and TT with their pair-matched controls. In addition, conditional logistic regression (CLR) analysis for disease association was performed. Conditional logistic regression is the analysis of choice for dichotomous paired data (in our case paired cases and controls) where the risk estimates associated with specific attributes (e.g genotype) are required to be adjusted for potential confounders. Reference genotypes were selected to be those that were common in our study population. A CLR test for trend in the odds ratios (OR) was performed to examine dose response effects in the relationship between genotype and disease. All analyses were performed using STATA (v8.0) software.

## Results

### Single-marker analysis

**Figure 1:**
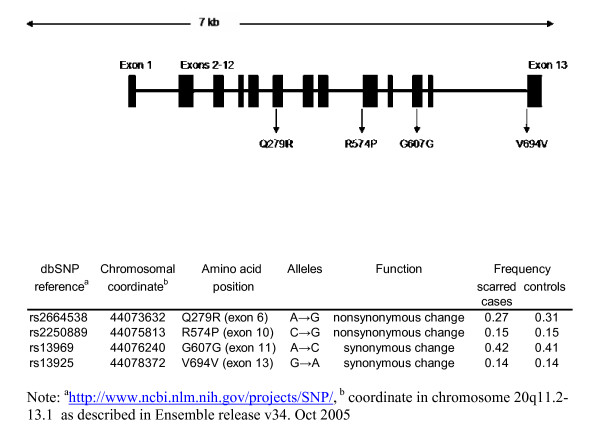
Diagram showing genotyped SNPs labelled according to the amino acid position.

A total of four exonic SNPs of population frequency > 10% were typed at the MMP9 locus (Figure 1). The distribution of all marker genotypes among cases and controls were in Hardy-Weinberg equilibrium (data not shown). Table 1 shows the genotype frequencies at each locus for each phenotype and the results of CLR association analysis of matched case-control pairs. The Q279R G allele frequency was marginally higher among controls than scarred subjects (0.31 vs 0.27, OR = 0.854, 95%CI = 0.714, 1.021, p = 0.083). The Q279R G allele was present in 45.1% of TS subjects against 51.6% of controls, and in 38.7% of TT subjects compared to 52.0% of controls. In adjusted analysis, presence of the Q279R G allele was associated with a reduced risk of TS (OR = 0.74, 95%CI = 0.59, 0.94, p = 0.012), and with a greater decrease in risk for the more severe TT phenotype (OR = 0.66, 95%CI = 0.46, 0.94, p = 0.021). Although there was some evidence for a trend towards decreasing risk of TS and TT with increasing number of Q279R G alleles, it did not reach statistical significance (OR = 0.864, 95%CI = 0.727, 1.027 and OR = 0.832, 95%CI = 0.644, 1.074 for TS and TT respectively). Heterozygotes Q279R AG were at lower risk of both TS and TT (OR = 0.69, 95%CI = 0.54, 0.81, p = 0.004 and OR = 0.57, 95%CI = 0.38, 0.85 p = 0.006 respectively) (data in Table 1).

**Table 1:** Genotype frequencies for the polymorphisms at the *MMP9 *locus in the study population and risk estimates from CLR analysis of matched case-control pairs.

Polymorphisms at the MMP9 locus in the study population					
Genotype	# of genotype (freq)		crude M-H OR(95%CI)	CLR-OR (95%CI)	P value
	Scarring cases (TS)	controls			

Q279R AA	330(0.55)	311(0.48)	1.18 (1.00, 1.38)	reference	-
Q279R AG	209(0.35)	270(0.42)	0.80 (0.67, 0.96)	0.70(0.54, 0.89)	0.004
Q279R GG	62(0.10)	61(0.10)	1.07 (0.74, 1.55)	0.95(0.64,1.41)	0.805
					

R574P CC	450(0.73)	459(0.72)	0.98 (0.86, 1.12)	reference	-
R574P CG	155(0.25)	161(0.25)	0.96 (0.77, 1.20)	0.97(0.74, 1.28)	0.845
R574P GG	15(0.02)	14(0.02)	1.07 (0.52, 2.22)	1.07(0.50, 2.28)	0.857
					

G607G AA	204(0.35)	193(0.35)	1.06 (0.87, 1.29)	reference	-
G607G AC	265(0.46)	268(0.48)	0.99 (0.83, 1.17)	0.91(0.68, 1.22)	0.538
G607G CC	114(0.20)	94(0.17)	1.21 (0.92, 1.59)	1.06(0.73, 1.55)	0.746
					

V694V GA	453(0.75)	443(0.74)	1.02 (0.90, 1.17)	reference	-
V694V GG	138(0.23)	145(0.24)	0.95 (0.75, 1.20)	0.94(0.71, 1.26)	0.694
V694V AA	14(0.02)	11(0.02)	1.27 (0.58, 2.80)	1.11 (0.49, 2.48)	0.807

Risk estimates from CLR analysis of matched case-control pairs					
					

Q279R AA	166(0.61)	131(0.48)	1.28 (1.01, 1.59)	reference	-
Q279R AG	75(0.28)	117(0.43)	0.64 (0.48, 0.86)	0.57 (0.38, 0.85)	0.006
Q279R GG	30(0.11)	25(0.11)	1.20 (0.71, 2.04)	0.95 (0.54, 1.69)	0.945
					

R574P CC	218(0.74)	213(0.71)	1.02 (0.85, 1.24)	reference	-
R574P CG	68(0.23)	80(0.27)	0.85 (0.62, 1.17)	0.76(0.50, 1.14)	0.181
R574P GG	7(0.02)	7(0.02)	1.00 (0.35, 2.85)	1.06(0.31–3.69)	0.927
					

G607G AA	97(0.35)	101(0.37)	0.96 (0.73, 1.27)	reference	-
G607G AC	127(0.46)	132(0.48)	0.96 (0.75, 1.23)	0.96(0.63, 1.46)	0.851
G607G CC	55(0.20)	43(0.16)	1.28 (0.86, 1.91)	1.03(0.60, 1.78)	0.908
					

V694V GA	225(0.79)	213(0.76)	1.06 (0.88, 1.27)	reference	-
V694V GG	54(0.19)	63(0.22)	0.86 (0.60, 1.23)	0.94(0.60, 1.48)	0.371
V694V AA	5(0.02)	5(0.02)	1.00 (0.29, 3.45)	0.79 (0.21, 2.95)	0.725

### Haplotype analysis

Table 2 shows the estimated frequency and associated risks of TS and TT by multivariate CLR analysis for inferred haplotypes at the *MMP9 *locus spanning exons 6 to 13. The 4 SNP sites segregated into seven haplotypes. Four common haplotypes with population frequency > 10% accounted for more than 80% of total variation, suggesting high linkage disequilibrium (LD) between segregating sites.

**Table 2:** Comparison of MMP9 haplotype frequency estimates in cases and controls and risk estimates from CLR analysis of matched case-control pairs. The haplotype configuration is as follows: Q279R, R574P, G607G and V694V.

haplotype	# controls	freq	# cases (TS)	freq	p-value	CLR:OR (95%CI)	# controls	freq	# cases(TT)	freq	p-value	CLR:OR (95%CI)
ACCG	450	0.34	466	0.36	-	reference	204	0.32	218	0.36	-	reference
ACAG	298	0.22	303	0.23	0.845	0.98(0.80, 1.21)	150	0.24	167	0.27	0.514	1.11 (0.82, 1.50)
GCAA	201	0.15	186	0.14	0.257	0.87 (0.69, 1.11)	91	0.14	75	0.12	0.224	0.79 (0.54, 1.15)
GCAG	161	0.12	130	0.10	0.048	0.77 (0.59, 0.99)	80	0.13	55	0.09	0.021	0.62 (0.41, 0.93)
AGAG	123	0.09	130	0.10	0.705	1.06 (0.79,1.40)	61	0.10	55	0.09	0.451	0.85 (0.56, 1.30)
AGCG	66	0.05	55	0.04	0.204	0.78 (0.53, 1.15)	33	0.05	27	0.04	0.171	0.66 (0.37, 1.20)
GCCG	30	0.02	32	0.02	0.977	0.99 (0.58, 1.68)	13	0.02	17	0.03	0.604	1.24 (0.56, 2.75)

The GCAG haplotype was associated with lower risk of TS and TT (Table 2). The risk of both TS and TT decreased with the number of copies of the haplotype GCAG (test for trend OR = 0.81, 95%CI = 0.63, 1.02, p = 0.07 and OR = 0.67 95%CI = 0.46, 0.96, p = 0.03 for TS and TT respectively). The GCAG-haplotype effect on risk of TT was greater than that on risk of TS.

Haplotypes containing the Q279R G allele, itself associated with decreased risk, were more commonly seen among controls than cases, although the difference in haplotype frequency between cases and controls reach significance only for haplotype GCAG. Conversely, the commonest haplotypes ACCG and ACAG were more frequently seen among cases than controls, and both carry the Q279R A allele. The strength of the single marker association is very similar to, if not greater than that of the haplotype, which supports the suggestion that the Q279R SNP itself may be causal.

## Discussion

The present study found a coding SNP (Q279R in exon 6) within the *MMP9 *gene to be associated with a lowered risk of trachomatous scarring and trichiasis, which was more marked for trichiasis. This supports the validity of the association because trichiasis is a more advanced form of cicatricial trachoma and also less subject to clinical misclassification than scarring trachoma.

The Q279R mutation leads to the substitution of a positively charged amino-acid (arginine) by an uncharged amino acid (glutamine) at position 279 within the *MMP9 *active site. Although the functional impact of this polymorphism on the protein is unknown, this variant is noteworthy; it is located in the coding sequence of one of the highly conserved fibronectin type II-like repeats which confers MMP-9 with high affinity binding to type IV collagen, type I gelatin and elastin [11,12]. The digestion of type IV collagen in the epithelial basement membrane has been suggested to be a key regulatory event in the initiation of fibrosis. A plausible explanation of these results is that Q279R represents a partial loss-of-function mutation within the proteinase whose presence reduces the development of fibrosis; these findings implicate MMP-9 as a key molecule in the pathogenesis of conjunctival scarring in trachoma.

The effect observed in this work was primarily associated with the Q279R GA heterozygous genotype. It is possible that the apparent lack of statistical effect of the GG homozygous genotype may have resulted from the modest sample size within the Q279R GG stratum. However we do not rule out the possibility of this finding being genuine and of biological significance; the increased risk of cicatricial trachoma associated with the Q279R AA homozygous genotype may be linked to the excessive MMP-9-driven proteolytic activation seen in the chronic inflammatory environment of the conjunctiva of subjects with trachoma[3,4]. Conversely, if Q279R GG homozygotes secrete only "sub-functional" copies of the enzyme, associated with low MMP-9 activity, this may result in disordered tissue remodelling because of a greater deposition of matrix, which has been found to correlate with hypertrophic scars[13]. In this setting, an advantage for Q279R GA heterozygotes could arise from the secretion of both native and defective forms of the enzyme at the site of inflammation upon cellular activation. This may lead to both a reduction of tissue proteolysis in chronic inflammation and protection against excessive tissue destruction and scarring.

MMP-9 expression and activity is up-regulated in the inflamed conjunctiva of trachoma subjects and increases with the severity of clinical inflammation [3,4]. There is evidence to suggest that the elevated local levels of TNF and IL-1β associated with chlamydial infection and inflammation[3] act as an important trigger of matrix degradation by inducing MMP-9 activity in inflamed tissue. Destruction of the ECM may facilitate cell migration and result in the characteristic infiltrating leukocytes and stromal cells (e.g. macrophages and fibroblasts) seen in trachoma subjects[4]. The secretion of MMP-9, cytokines and chemokines by these cells may drive the inflammatory process into a positive feedback loop. MMP-9 can activate pro-inflammatory cytokines[14] which may further increase and perpetuate inflammation leading to persistent tissue damage. Later during the tissue repair phase, excessive MMP activities may contribute to contractile scarring, characteristic of TS and TT, through their role in ocular fibroblast-mediated matrix contraction [15,16]. It is plausible that persistently increased MMP9 activity in the conjunctiva of subjects with trachoma may be a key event in the pathogenesis of conjunctival scarring through excessive degradation of ECM and fibrosis. The recent characterisation of MMP-9 expression and activity in mouse strains exhibiting variable susceptibility to sequelae of genital *Chlamydia muridarum *(MoPn) infection[5] supports this suggestion: C3H/HeN mice, which are particularly susceptible to fibrotic sequelae in this model[6], exhibited greater MMP-9 transcription and activity during infection.

The blinding lesion in trachoma is corneal opacity (CO), which is thought to result from the lashes rubbing against the eyeball (TT) and damaging the cornea. Epidemiological studies indicate that after successful TT surgery, CO can develop[17], and there is evidence that inflammatory episodes in TT subjects correlate with the presence of CO[15]. MMPs are expressed at sites of epithelial loss in ulcerated corneas and found in the tear film of patients with external ocular inflammatory disorders[18]. In the cornea, an imbalance in MMP9 production has been implicated in corneal scarring and loss of corneal transparency and visual function[7]. Proteolytic destruction of the glandular tissue contributes to reduced secretion of tears, and is associated with a number of corneal pathologies[7]. The constant exposure of the corneal surface to conjunctival and/or tear MMP-9 and inflammatory cytokines may contribute to progressive corneal opacification. CO may be a relevant phenotype for *MMP9 *genetic association analysis, but for practical reasons large numbers of patients with CO are difficult to come by.

## Conclusion

This work supports the hypothesis that MMP-9 has a role in the pathogenesis of blinding trachoma. The risk reductions associated with the Q279R exonic mutation are modest: 26% for scarring and 34% for trichiasis. CLR analysis suggests that these effects are independent of those previously reported in the interleukin 10 (*IL10*) and interferon gamma (*IFNγ*) loci [10]. This is consistent with a model of cicatricial trachoma as a complex disorder with multiple genetic factors contributing to risk of scarring and trichiasis after ocular chlamydial infection. Further studies of MMP-9 in chlamydial infection and of the functional significance of genetic variation at the MMP-9 locus, are warranted.

## Abbreviations

ECM -extracellular matrix, MMP -matrix metalloproteinase, SNP -single nucleotide polymorphisms, CLR -conditional logistic regression, TIMPs – tissue inhibitors of matrix metalloproteinases, WHO -World Health Organization, TS -scarring trachoma, TT -trichiasis, OR -odds ratio, CI -confidence interval, LD- linkage disequilibrium, CO -corneal opacity, TNF -tumor necrosis factor, IL-1β-interleukin 1 beta, IL10 -interleukin 10, IFNγ-interferon gamma.

## Competing interests

The author(s) declare that they have no competing interests.

## Authors' contributions

AN collected, edited, analysed the data and wrote the manuscript. RB directed the study, study design and co-wrote the manuscript. GC study design and contributed to the manuscript. DM, MH and MB directed the study, study design and contributed to the manuscript. DK and KR co-directed the study. OJ and HJ collected clinical data and provide clinical material

## Pre-publication history

The pre-publication history for this paper can be accessed here:


